# Selenium targets resistance biomarkers enhancing efficacy while reducing toxicity of anti-cancer drugs: preclinical and clinical development

**DOI:** 10.18632/oncotarget.24297

**Published:** 2018-01-23

**Authors:** Yousef Zakharia, Arup Bhattacharya, Youcef M. Rustum

**Affiliations:** ^1^ University of Iowa Division of Medical Oncology and Hematology, Holden Comprehensive Cancer Center, Iowa City, IA, USA; ^2^ Roswell Park Cancer Institute, Department of Pharmacology and Therapeutics, Buffalo, NY, USA

**Keywords:** selenium, HIFS, translational medical research, transcription factors, microRNAs

## Abstract

Selenium (Se)-containing molecules exert antioxidant properties and modulate targets associated with tumor growth, metastasis, angiogenesis, and drug resistance. Prevention clinical trials with low-dose supplementation of different types of Se molecules have yielded conflicting results. Utilizing several xenograft models, we earlier reported that the enhanced antitumor activity of various chemotherapeutic agents by selenomethione and Se-methylselenocysteine in several human tumor xenografts is highly dose- and schedule-dependent. Further, Se pretreament offered selective protection of normal tissues from drug-induced toxicity, thereby allowing higher dosing than maximum tolerated doses.

These enhanced therapeutic effects were associated with inhibition of hypoxia-inducible factor 1- and 2-alpha (HIF1α, HIF2α) protein, nuclear factor (erythyroid-derived 2)-like 2 (Nrf2) and pair-related homeobox-1 (Prx1) transcription factors, downregulation of oncogenic- and upregulation of tumor suppressor miRNAs. This review provides: 1) a brief update of clinical prevention trials with Se; 2) advances in the use of specific types, doses, and schedules of Se that selectively modulate antitumor activity and toxicity of anti-cancer drugs; 3) identification of targets selectively modulated by Se; 4) plasma and tumor tissue Se levels achieved after oral administration of Se in xenograft models and cancer patients; 5) development of a phase 1 clinical trial with escalating doses of orally administered selenomethionine in sequential combination with axitinib to patients with advanced clear cell renal cell carcinoma; and 6) clinical prospects for future therapeutic use of Se in combination with anticancer drugs.

## INTRODUCTION

The human tumor microenvironment of non-malignant cells and stroma is molecularly and immunologically heterogeneous. It is now abundantly clear that growth, metastasis and therapeutic response is regulated by multiple interactive pathways that make modulating specific targets necessary but which by itself is not always sufficient in advancing long-term survival and remission in cancer patients. With better understanding of various mechanisms in cancer biology and recent progress in molecular and immunological targeted therapies, there have been some significant advances in treatment outcome. Yet durable responses are achieved only in a small fraction of patients with specific types of cancers, such as melanoma, lung, and renal.

To build upon the knowledge gained and the clinical advances achieved to date, it is imperative that mechanism-based combinations of targeted and cytotoxic agents be implemented and validated clinically with a focus on dose, sequence, route and duration of administration. The focus of this review is to identify small molecules that can effectively and selectively modulate the *in vivo* expression of biomarkers commonly expressed in a majority of cancers that are implicated in angiogenesis and drug resistance. Hypoxia-inducible and constitutively-expressed 1α and 2α (HIFs) factors and vascular endothelial growth factor (VEGF) have been shown to be targets of methylselenocysteine (Se-methylselenocysteine [MSC]) and selenomethionine (SLM) in xenograft models [1–19]. Therapeutic doses of MSC have been found to downregulate expression of oncogenic miRNAs, and upregulate tumor suppressor miRNAs in clear cell renal cell carcinoma (ccRCC) and xenografts. This modulation of biomarkers by SLM or MSC was associated with an enhanced antitumor activity of a wide range of anticancer drugs [1, 3–8, 13–19].

Selenium (Se) is a trace element present in high concentrations in Brazil nuts [20, 21], fish, and in plants grown in soil with high Se content. Se is classified as an antioxidant that regulates cell metabolism, oxidative stress, and DNA- and RNA-protein-synthesis. Se exists either in organic forms such as SLM and MSC, or in inorganic forms such as selenide and selenite (Table 1) [22]. Inorganic Se is converted in plants to organic Se, and is retained as such in animals and humans. The daily recommended dose for adults is approximately 50μg. The circulating Se blood levels in adult populations worldwide vary considerably, influenced by dietary and supplement intake, and Se soil levels where consumable vegetation is grown. Published reports suggest an inverse relationship between Se status and risk for colon, prostate, lung, and bladder cancer, among others [23–26]. Se toxicity has been reported in patients with Se intake approximately 200 times the doses used in prevention trials, and at least 70 times the SLM doses used in therapeutic trials [27, 28]

**Table 1:** Selenium compounds for cancer prevention and therapy

Forms of Se	Active metabolites	Remarks
Sodium selenite	Hydrogen selenide	50% absorbed and retained. High toxicity – genotoxic, induces single strand DNA breaks *in vitro*. Conversion to methylselenol is a rate-limiting step and occurs when selenite is present in excess, selenite do not regulate the expression of NKG2D ligand that trigger immune activation [22]
Sodium selenate	Hydrogen selenide	Low toxicity - almost completely absorbed but most gets excreted in urine before being incorporated into protein. Activator of PP2A phosphatase
Methyl selininic acid	Methyl selenol	High toxicity with low dose tolerance and of little nutritional value. Regulates expression of NKG2D ligand that trigger immune activation [22]
Selenocysteine	Hydrogen selenide, Methyl selenol	Toxic in higher concentration similar to selenite [57]. Chemically unstable and difficult to handle.
Selenomethionine	Methyl selenol Hydrogen selenite,	Well tolerated, low toxicity but binds to plasma components. Multiple step conversion to methylselenol. Hydrogen selenite conversion into methylselenol is a rate-limiting step and occurs only when selenite is present in excess [22]
Methylselenocysteine	Methyl selenol	90% absorbed, accumulates in a free pool post ingestion. Lowest toxicity amongst Se compounds and more bioactive making it the most ideal candidate for supplementation and therapeutic usage. Bind relatively less to plasma components. A single step conversion β-lyase leads to methylselenol. It regulate the expression of NKG2D ligand that trigger immune activation [22]

The aim of this review is to identify targets associated with the observed therapeutic benefit achieved by the sequential combination of SLM or MSC with anticancer drugs to provide the basis for expanded preclinical and clinical therapeutic use.

### Chemical structure of investigated se

**Figure 1:**
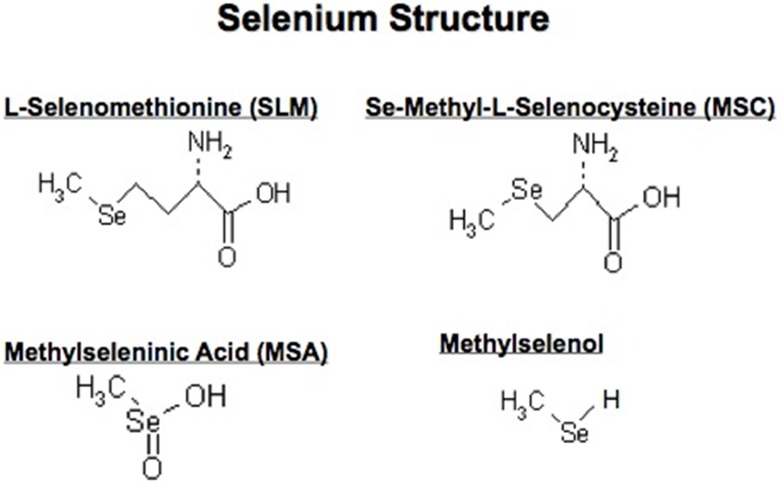
Chemical structure of L-Selenomethionine, Se-methyl-L-selenocysteine (MSC), methylseleninic acid (MSA) and methylselenol

The biochemistry and functional role of Se has been extensively reviewed [20–58]. Briefly, Se-containing molecules have been found to exert pleiotropic effects against multiple targets associated with tumor growth, metastasis and drug resistance. The chemical structures of SLM and MSC used in *in vivo* studies and their presumed active metabolite, MSA, used in *in vitro* studies, are shown in Figure 1. SLM and MSC are antioxidant pro-drugs with relatively low *in vitro* cytotoxic effects and are activated *in vivo* to the active moiety methylselenol by β-lyase. While SLM requires multiple enzymatic activations, MSC has one-step activation, is less toxic and less protein bound. Sodium selenite and selenized yeast are molecules metabolized to selenide with subsequent conversion to methylselenol (Table 1) [31]. Methylselenol derived from MSC, but not selenide derived from SLM, has been reported to regulate the expression of ligands that trigger immune activation [30, 31]. Differences in the structural-based metabolic activation of Se are likely contributors to the differences in their mechanisms of action and efficacy in prevention and therapeutic trials. There is an inverse correlation between the basal-levels of plasma Se and cancer incidence and mortality [41, 59]. Investigators at Pennsylvania State University are currently developing interesting and potentially promising Se-containing molecules [42]. Selenocompounds have been reported to exert epigenetic effects, in part by interfering with the one-carbon metabolism that provides the methyl donor for DNA methylation [58, 60].

The importance of Se as a nutritional supplement and its’ potential as a chemopreventive agent have also been evaluated [30, 31]. In contrast, the therapeutic potential of specific types of Se, their doses, schedule and sequence in the selective modulation of antitumor activity *in vivo* of standard and newly developed targeted agents in advanced cancers has not yet been fully addressed.

### Se-containing molecules in cancer prevention

A number of clinical prevention trials have utilized SLM, sodium selenite, and selenized yeast [34, 37, 38]. Table 1 summarizes the different forms of Se that have been evaluated in *in vitro* and *in vivo* models. SLM is better absorbed than selenite, and plasma concentrations derived from SLM dosing are significantly higher than those derived from the other Se molecules, with no dose-dependent modulation of glutathione peroxidase or selenoprotein P1 [33]. The low doses of SLM used in the SELECT trials [36, 40] and sodium selenite and selenized yeast used in other prevention trials were based on an initial trial conducted over 30 years ago by Clark et al [35]. Recently, the use of SLM as a cancer preventive agent in the SELECT trial did not demonstrate favorable clinical results [36, 40].

It is reasonable to assume that the lack of demonstrable clinical benefit is multifactorial and could include the type of Se used. SLM, rather than high-selenium brewer's yeast with more than 20 Se-containing species used in other trials [35], the dose of Se administered; gender dependent metabolism of Se; pre-existing circulating basal levels of Se and uncontrolled use of other supplementary agents such as vitamins, non-steroidal anti-inflammatory drugs, and trace elements. For example, supplements with zinc which is needed for the maintenance of immune function, reportedly protect cells in the early steps of the apoptotic pathway [44]. Further, circulating levels of Se in certain population such as in New Zealand is lower than those in the western countries [32] and supplementation may benefit such population more than those with a higher baseline. In the SELECT trials, only men with high Se baseline level were enrolled. The SELECT and the New Zealand trials [35, 40] were conducted in male patients utilizing different types of Se. Our preclinical data confirms a greater therapeutic efficacy from MSC or SLM in combination with anticancer drugs than when used in combination with selenized yeast or MSA.

### Selective modulation of the antitumor activity and toxicity of anticancer drugs by MSC and SLM is dose and schedule dependent

**Figure 2:**
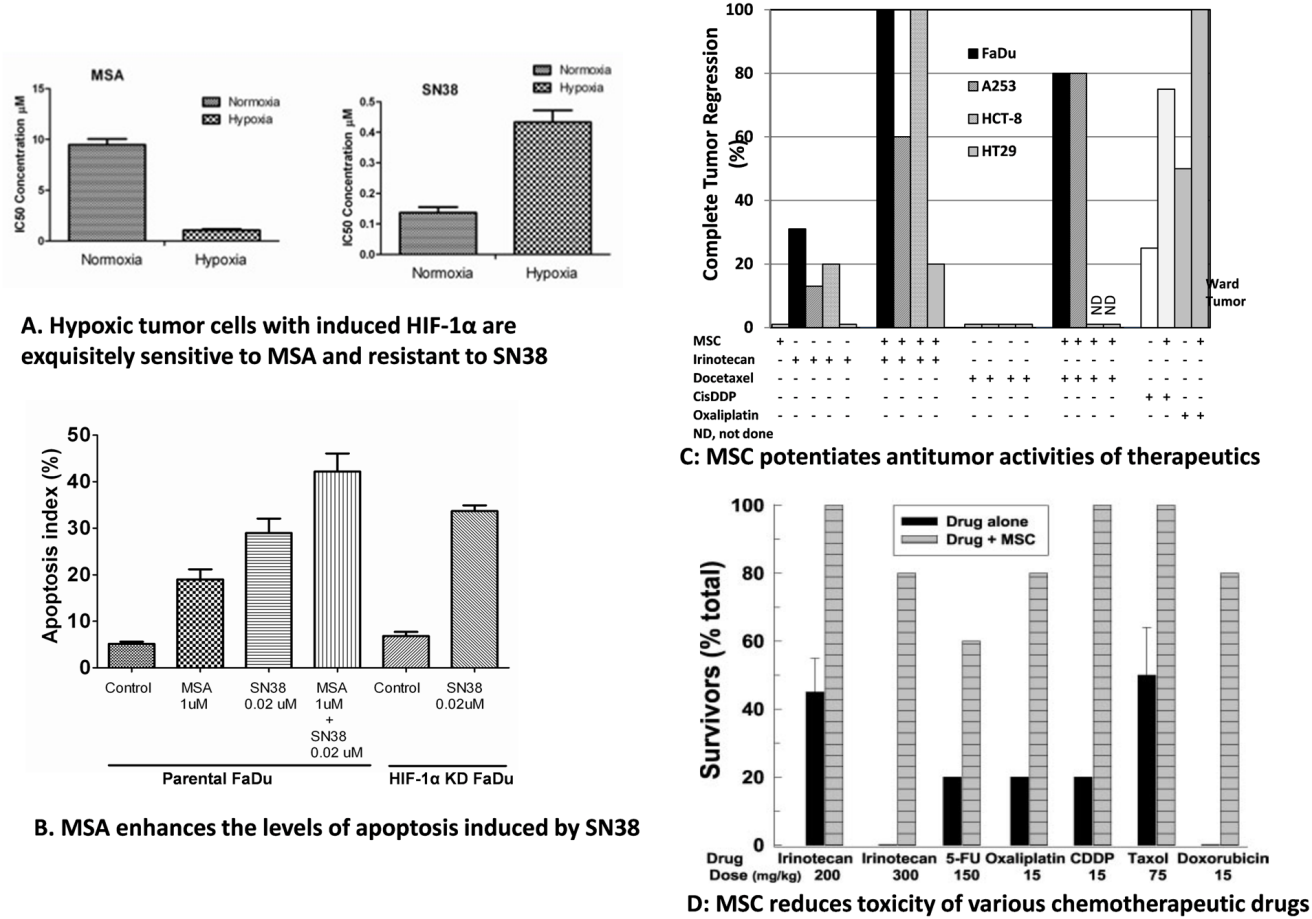
Differential *in vitro* effects of MSA and SN38 in hypoxic cells expressing HIF-1α and in normoxic cells with no detectable HIF1α protein expression **(A)**. Effects of MSA on the level of apoptosis induced by MSA and SN38 in hypoxic FaDu tumor cells expressing HIF1α, and, in HIF-1α shRNA knockdown FaDu cells treated with SN38 alone **(B)**. Effects of MSC on the antitumor activity of anticancer drugs in head and neck FaDu and A253; colorectal HCT8 and HT29 xenografts, and, in rats bearing Ward colon tumors **(C)**. Effects of MSC on the toxicity induced by lethal doses of anticancer drugs **(D)**. CR, complete tumor response with no evidence of tumor relapse at 3 months.

Studies in FaDu head and neck squamous cell carcinoma cells expressing hypoxia-inducible HIF1α confirmed that HIF is a Se target and its downregulation enhances drug effects. Under normoxia, FaDu cells with no detectable HIF1α protein are relatively less sensitive to MSA, the active metabolite of MSC and SLM, but more sensitive to SN38, the active metabolite of irinotecan (Figure 2A). In contrast, under hypoxia, FaDu cells expressing HIF1α protein become exquisitely more sensitive to MSA, but less sensitive to SN38. The observed enhanced SN38 cytotoxicity by MSA is associated with a pronounced inhibition of HIF1α and an enhanced level of apoptosis induced by SN38 (Figure 2B). Thus, chemoresistance of FaDu tumor cells expressing HIF1α to SN38 can be reversed as a consequence of HIF1α inhibition by MSA.

Saifo et al demonstrated that β-catenin, a molecule associated with drug resistance, is a target of Se and its inhibition is associated with increased multiple drug cytotoxicity [13]. Further, degradation of β-catenin induced by GSK-3β phosphorylation is not a general mechanism but is cell type-dependent [61]. Studies in prostate tumor cell lines have demonstrated that growth of hormone refractory prostate cancer cells can be controlled by treatment with MSA and these effects are associated with the downregulation of HIF-1α, possibly through stabilization and/or increase in prolyl hydroxylase activity, and with the downregulation of VEGF, and Glut1. Beppu et al [62] and Puppo et al [63] reported that inhibition of HIFs by low doses of topotecan resulted in dose-dependent inhibition of VEGF in neuroblastoma tumor cells. Recently, Sun et al also demonstrated that Se-enriched extracts from pyracantha fortuneana inhibit the growth and proliferation of ovarian cancer cell lines by enhancing apoptosis and inhibiting β-catenin signaling [54] which has been found to be associated with transcriptional upregulation of HIF-1α [64] and colorectal tumorigenesis [65].

The role of Se in the *in vivo* modulation of anticancer drugs has been evaluated in several human tumor xenografts, including head and neck FaDu and A253, colorectal HT29 and HCT8 models, ward rat colon tumors (Figure 2C) and 786.0 ccRCC xenografts [19]. The observed therapeutic augmentation between Se and anticancer drugs was associated with PHD-dependent and VHL-independent degradation of HIFs, downregulation of cyclooxygenase-2, and nitric oxide synthase, with enhanced pericyte recruitment and vascular normalization [3, 5, 16, 18]. In contrast to the relatively poorly differentiated FaDu and HCT8 xenografts which were sensitive to the MTD dose of irinotecan, the well-differentiated tumors of A253 and HT-29 xenografts were relatively resistant (Figure 2C). Optimal efficacy was achieved only when MSC was administered in sequential combination with two to three times the MTD of irinotecan, a dose that is lethal but can be administered safely in combination with MSC [15]. Thus, protection of normal tissues by MSC against treatment-induced toxicities allows drug dose administration higher than their respective MTDs. This therapeutic outcome from MSC and anticancer drugs administered in combination setting in several preclinical xenografts was achieved only when MSC was orally administered daily for a minimum of seven days prior to and continued daily during the duration of anticancer drug treatment. Concurrent treatment, i.e., without MSC pretreatment, did not achieve similar therapeutic outcome. The MSC or SLM dose administered to achieve a plasma Se concentration comparable to those achieved with SLM dose during SELECT prevention trial was insufficient in modulating anti-cancer efficacy of chemotherapeutic drugs in the same xenorgrafts. Similar therapeutic effects have been reported between MSC and VEGF/VEGFR targeted agents in combination with chemotherapy [16, 19].

The potential role of MSC and SLM in protecting normal tissues from drug-induced toxicity has been evaluated in xenografts (Figure 2D). Oral administration of 8 mg/kg Se offers selective protection against toxicity induced by lethal doses of various chemotherapeutic agents, with different mechanisms of action and different target organs. In these studies selective protection of normal mouse bone marrow and epithelial gut cells by SLM and its active metabolite MSA from cisplatin and radiation-induced toxicity was associated with an enhanced level of the XPC DNA repair gene [9, 12]. SLM induces XPC/Gadd45, thioredoxin reductase and p53 in normal bone marrow and gut epithelial cells that expresses wild type p53.

In summary, Se-containing molecules used *in vivo* at their optimal doses and schedules in sequential combination with varying chemotherapeutic agents are a highly selective and effective treatment resulting in durable cure in several tumor xenografts. Enhancement of antitumor activity and protection against drug-induced toxicity are unique properties of MSC and SLM that are defined by a therapeutically effective and non-toxic dose and schedule.

### Se targets

**Chart 1:**
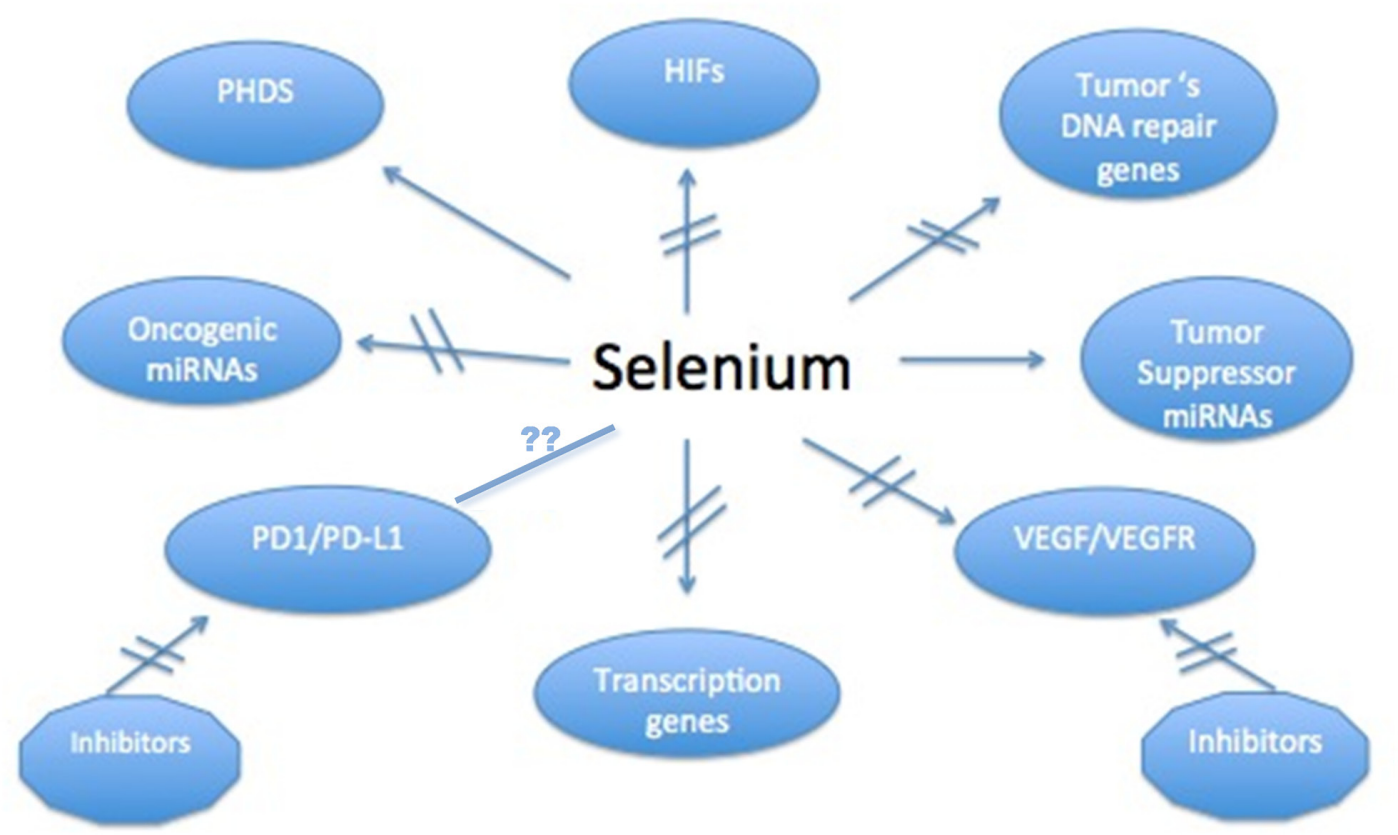
Proposed biomarkers modulated by therapeutic doses of MSC in several human tumor xenografts

Data generated in our laboratories and by others have demonstrated multiple targets affected by Se [1–19]. We have found that the observed enhanced therapeutic efficacy of anticancer drugs by Se is associated with a collective modulation of several targets found altered in preclinical models. However, it is unclear at this time whether such modulation of a single target by Se would be necessary and sufficient for tumor cell sensitization to anticancer drugs. It is likely, however, that the pleiotropic role of MSC and SLM in modulating multiple targets would be essential for the selective and effective sensitization of tumor cells to subsequent treatment with anticancer drugs. The potential Se targets presented in chart 1 are discussed below

#### HIF1α and 2α

HIFs as critical therapeutic targets have been extensively evaluated [43, 52, 53, 57, 64–96]. A study by Zhao et al [76] indicated that while there is homology between HIF1α and HIF2α, HIF2α might play an important role in the growth, metastasis and resistance to chemotherapy in digestive tract cancers. Further studies are required to confirm the role of HIF2α in tumor biology and as a predictor of drug response in digestive tract cancers and other solid tumors. HIF1α downregulates C-Myc and mTOR while activating P53 while HIF2α upregulates C-Myc and downregulates P53 and mTOR [69, 74]. In renal cancers, HIF1α and −2α seem to play an opposing role, with HIF1α acting as a tumor suppressor and HIF2α behaving as an oncogene [75]. A report by Imamura et al demonstrated the divergent roles of HIF1α and HIF2α [72]. Cui et al found that HIF1α/2α overexpression in 244 pancreatic cancer specimens was associated with the activation of lactate dehydrogenase A [67].

Cellular accumulation of HIFs are determined by the rate of protein synthesis and its degradation. Under normoxia, oxygen-dependent hydroxylation of proline in HIFs by prolyl hydroxylases is a key step that leads to the recognition of HIFs by VHL protein, followed by its degradation through the ubiquitin-proteasome pathway. Therefore, under normoxia, prevalent in most normal organs, HIF protein is rapidly degraded and thus is undetectable. Under hypoxia, the enzymatic activity of PHDs decreases, resulting in a decreased hydroxylation and degradation of HIFs. Unlike in many solid tumors, ccRCC tumors, at initial diagnosis, are normoxic, highly vascular and express high levels of HIFs [8, 16, 17]. With the recognition that HIFs are upregulated by both hypoxia-dependent and independent pathways and play a crucial role in dug resistance and angiogenesis, a number of HIFs inhibitors are currently under clinical development. However, many of these agents, such as topotecan, are toxic and offer limited efficacy [92, 97–100].

As an alternative approach, our laboratory was the first to demonstrate that therapeutic doses and schedules of MSC and SLM are potent and selective *in vivo* inhibitors of hypoxia-induced and constitutively-expressed HIFs [8, 16]. However, the efficacy of HIF-targeted agents is likely to be dependent on its ability to reach the intended targets in sufficient enough concentrations in tumor in order to lower the levels and function of the intended targets while the incidence, intensity, and distribution of the targets are also equally critical. For example, in treatment of ccRCC xenografts that are often characterized by a VHL loss of function and results in constitutive expression of HIF1α and HIF2α [66], therapeutic doses of MSC caused a partial downregulation of HIFs with limited antitumor activity. Enhanced inhibition of HIF by MSC in combination with topotecan, an inhibitor of HIFs synthesis, results in a significant increase in treatment efficacy. In contrast, treatment with therapeutic doses of MSC alone (without topotecan) of colorectal and head and neck xenografts expressing lower incidence of HIFs resulted in a pronounced inhibition of HIF1α and sensitizes cancer cells to treatment with a variety of anticancer drugs [8, 16].

#### HIF1α regulated VEGF

Studies in our laboratory have demonstrated that HIF1α and HIF2α proteins are overexpressed in 92% of primary ccRCC, 27% in colorectal, and 38% in head and neck tumors [16, 17]. In contrast, VEGF-A was expressed in 54%, 79%, and 97% of ccRCC, colorectal, and, head and neck tumors, respectively. The average immunoscore of VEGF-A in ccRCC, colorectal, and head and neck tumors were 2.3, 5.7, and 4.2, respectively [16, 17]. ccRCC is known to respond to VEGF-targeted agents, but colorectal or head and neck cancers do not. Since the dose and schedule of the VEGF-targeted agents are generally kept constant irrespective of cancer types, it is possible that the documented responses to VEGF/VEGFR inhibitors of ccRCC patients are due, in part, to the lower levels of VEGF-A target agents. Our investigations have shown that levels of VEGF-A are higher in tumor cells expressing HIF1α than in cells expressing HIF2α [16, 18]. In *in vivo* studies, therapeutic doses of MSC partially downregulated VEGF-A in cells expressing HIF1α but not in cells expressing HIF2α. Significant levels of VEGF remained stably expressed even though HIF1α and HIF2α were similarly inhibited by MSC. These results suggest that VEGF levels are differentially regulated by HIF1α and HIF2α, and a complete inhibition of VEGF may require administration of a combination of agents that inhibit VEGF regulated by HIF1α and those that target VEGF-regulated by HIF-independent mechanisms. This concept was confirmed by use of MSC, an enhancer of HIF degradation, in sequential combination with topotecan, a cytotoxic anticancer drug that also inhibits HIF synthesis, and, sunitnib, an inhibitor of VEGFR [19]. Validation of this concept in additional relevant preclinical models with similar targets of interest could provide the basis for clinical translation of this approach.

#### Tumor microenvironment (TME)

TME is comprised of heterogeneous cell types, including stromal cells, tumor infiltrating lymphocytes, macrophages, and multiple immune cells [101–110]. TME regulates neoangiogenesis, cancer metastasis, and response to anti-cancer treatments. Stromal-modulating growth factors act in a paracrine fashion to disrupt normal tissue homeostasis such as neoangiogenesis and inflammatory responses. Se is known to modulate expression levels and function of immune cells within the TME milieu [5]. Unstable, chaotic and immature tumor vasculature leads to heterogeneity in tumor blood flow, a higher tumor interstitial fluid pressure (10-100 mm Hg compared to around zero in normal tissue), tumor hypoxia and a low extracellular pH within TME. Along with the presence of extracellular matrix such as collagen and mucin within TME, this contributes to a poor drug delivery while facilitating clonal selection of resistant cancer cells [1, 5, 103, 104]. Unlike normal tissues, TME is unstable with leaky vasculature, and expresses multiple molecular and immunological biomarkers associated with angiogenesis and drug resistance [102, 103, 106, 108]. Cells comprising TME support growth and development of cancer cells while representing a potential barrier to intratumoral drug delivery in effective cytotoxic concentrations sufficient enough for tumor cell kill. TME is, therefore, recognized as a potential target for drug and therapy development. TME stabilization may be achieved by treatment with agents that selectively target drug resistance biomarkers. Thus, if tumor cells are the ultimate target, TME is the initial gatekeeper modulating drug delivery and response.

**Figure 3:**
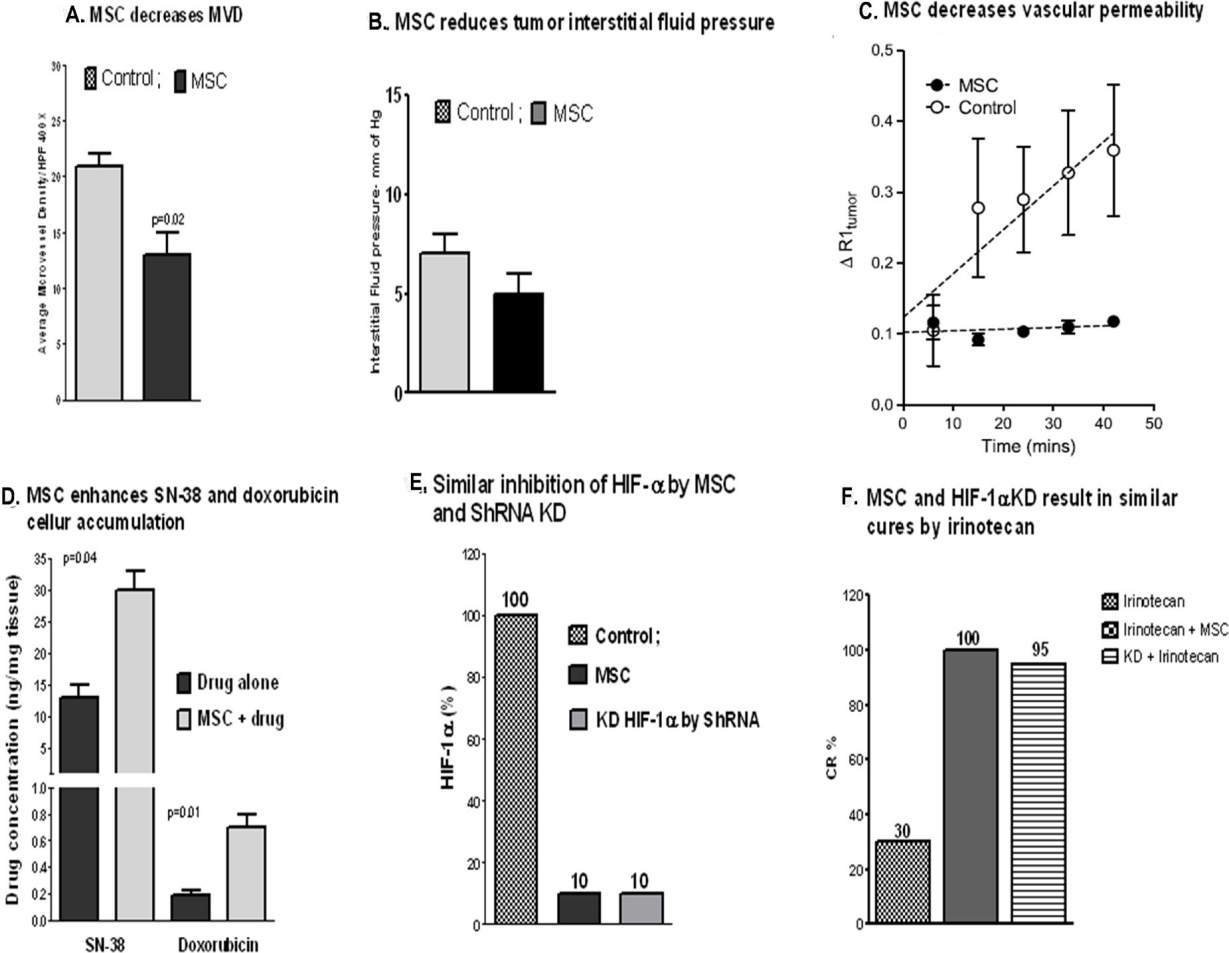
Pleiotropic effects of MSC Effects of MSC on TME in FaDu xenografts: a reduced microvessel density (**A**, MVD) along with a reduced tumor interstitial pressure **(B)** and an increased vessel normalization effect as reflected by a reduced vascular permeability [3] compared with the untreated **(C)** resulting in an increased drug accumulation in tumors **(D)**. Inhibition of HIF-1α by MSC was similar to the one seen with HIF-1α knockdown ShRNA **(E)** and the effect of MSC in the combination therapy with irinotecan mimicked the treatment response seen with irinotecan and HIF1α knockdown shRNA **(F)** [8].

The potential *in vivo* role of therapeutic doses and schedules of various forms of Se in targeting tumor cells and surrounding TME has not been extensively reported. Our group was the first to report that therapeutic doses of MSC cause an antivascular effect, leading to decreased microvessel density, lower tumor interstitial fluid pressure, optimal vascular permeability through an increased pericyte coverage of blood vessels (or vessel normalization), and a selective increase in drug distribution within tumors (Figure 3). The antiangiogenic effects of MSC are similar to that of bevacizumab in human cancer cells [103, 111]. Figure 3A-3D depicts the antivascular effects of therapeutic doses of MSC, resulting in enhanced intratumoral drug accumulation. These effects followed an inhibition of HIF1α and led to an enhancement of the antitumor activity of irinotecan (Figure 3E, 3F). Therapeutic augmentation was achieved only when irinotecan was administered after optimal inhibition of HIF1α and stabilization of TME by MSC. Paolicchi et al demonstrated that activation of HIF1α in the hypoxic TME causes activation of gene regulating glucose transport, glycolysis, angiogenesis, and changes in mitochondrial functions [71]. A study by Ribiero and Okamoto supported the concept that tumor vascular pericyte coverage contributes to TME stabilization [108].

Although current data suggests that HIF is a critical therapeutic target of MSC, observed TME stabilization and enhanced efficacy of anticancer drugs are likely due to additional effects by Se on other targets. This may include altered expressions of miRNAs known to play a key role in the crosstalk between TME and tumor cells.

#### DNA repair and transcription genes

Induction and maintenance of DNA double-strand break is generally associated with positive tumor response to therapy. In contrast, repair of DNA damage is associated with therapeutic resistance. Tumors that overexpress HIF are associated with a higher expression of miRNAs such as miRNA-210, which in turn regulates factors implicated in DNA repair pathways [70, 112]. Smith et al demonstrated in mouse bone marrow cells and human head and neck cancer cell lines A253 and FaDu that while Se offered a selective protective effect against drug-induced DNA damage in normal bone marrow cells, not in tumor cells. Se was found to selectively upregulate XPC, a DNA repair gene, in normal cells but not in tumor cells. The protective effects of Se were p53-dependent [9, 14] and the observed DNA damage involved a Ref1/p53/Brca1 protein complex. Collectively, these effects of MSC were associated with an enhanced efficacy of radiation and antitumor activity of cisplatin.

Peroxiredoxin-1 (Prx1) is frequently elevated in many solid tumors and TME and is often associated with tumor growth and drug resistance. Transcription factor Nrf2, over-expressed in many human solid tumors, also reportedly regulates miRNA-155 expression [113, 114]. Increased translation of Nrf2 leads to an upregulation of Prx1. These transcription factors play a dual role in that they protect normal tissues against oxidative damage while promoting tumor growth, metastasis, and chemoresistance. Park et al used the NCI-60 panel of NSCLC cell lines to demonstrate Prx1 over-expression in nine non-small cell lung cancer (NSCLC) cells [10, 11]. Data generated by Kim and others suggests that Nrf2/Prx1, which are upregulated in primary human lung cancer, are potentially critical therapeutic targets [11] (Table 2). Therapeutic doses of Se can selectively downregulate Nrf2/Prx1 levels induced by hypoxia in tumor cells while up-regulating these markers in several normal mouse tissues [11]. Thus, selective inhibition of transcription factors by MSC was found to enhance antitumor activity of chemo and radiation therapies in lung cancer A549 and colorectal cancer HT29 while protecting the normal healthy tissues.

**Table 2:** MSC differentially activate Nrf2 in A549 lung tumor vs normal lung tissue [11]

	Relative Intensity			
	TUMOR		LUNG	
	C	MSC	C	MSC
Prx1	5.4	1.2	1.2	3.8
Nrf2	6.0	1.0	1.0	4.0

In brief, therapeutic doses of MSC are effective and selective inhibitors of specific DNA repair gene and specific types of transcription factors. Modulation of these markers by MSC results in an enhanced antitumor activity of chemo and radiation therapies in preclinical models.

#### Altered oncogenic and tumor suppressor miRNA expression

miRNAs are single-stranded, small noncoding RNAs, approximately 22 nucleotides long, that function as post-transcriptional regulators of multiple genes. There are two types of altered miRNAs: 1) oncogenic miRNAs are upregulated in advanced cancers and in unstable TMEs, where they are associated with enhanced angiogenesis, metastasis and drug resistance; and, 2) tumor suppressor miRNAs, which are downregulated in tumor cells and TMEs while being linked to drug efficacy [116].

The altered expression of specific types of miRNAs is associated with therapeutic resistance and thus may serve as cancer biomarkers. Redova et al reported that in 35 ccRCC tumors, compared with 10 non-tumor kidney tissues, miRNA 210 and 155 levels were 5.99 and 4.57 fold higher, respectively [115]. miRNAs are master regulators of cell stemness, cancer metastasis and drug resistance [116]. miRNA 210 is inversely correlated with disease-free survival and overall survival of patients with NSCLC. miRNA 155 and 210 have been identified as VHL-regulated miRNA, activated by HIF1α and associated with drug resistance [117], and as prognostic and diagnostic biomarkers for overall survivor in glioblastoma following surgery [118]. miRNA 155 also represses a ubiquitin ligase that promotes degradation of NF-k-β family transcription factor c-Rel. An upregulated miRNA 155 modulates T-cell proliferation responses by targeting cytotoxic T lymphocyte-associated antigen 4 [119]. Based on published data, oncogenic miRNA 155 and 210 seem to function as pleiotropic regulators of immunologic and molecular biomarkers associated with therapeutic resistance in cancer cells.

In view of the identification of altered specific oncogenic and tumor suppressor miRNAs as potential therapeutic targets, it is a critical and unmet need to identify agents that can selectively and effectively target such miRNAs. Several approaches currently under evaluation are aimed at downregulating oncogenic miRNAs and/or upregulating tumor suppressor miRNAs. This includes chemotherapeutic drugs, antisense oligonucleotides and viruses [77, 94–96, 120]. To date, *in vivo* instability of TME, poor drug uptake, dose-limiting toxicity and the need for better evaluation of the effects of the clinical agent under development on miRNA targets with its consequences in therapeutic outcomes continue to represent a major challenge. Our published data indicates that effective dosing and scheduling of MSC and perhaps SLMs that are capable of inhibiting HIFs are also associated with the downregulation of 28 oncogenic miRNAs and the upregulation of 12 tumor suppressor miRNAs expressed in ccRCC xenografts. The miRNAs modulated by MSC include the oncogenic miRNA 155, 106b, and 210, and the tumor suppressor miRNA-Let7b, −85, and 328 which have also been found in primary ccRCC tumor biopsies.

In brief, altered expression of various miRNAs seem to play a pivotal role in the regulation of different immunological and molecular biomarkers implicated in tumor drug resistance and TME heterogeneity.

#### Immune response cells and immune response checkpoint

Programmed death 1 (PD-1), an immune inhibitory receptor expressed on several immune cells including cytotoxic T cells, interacts with two ligands—programmed death ligand (PD-L) 1 and PD-L2. PD-L2 is expressed primarily on macrophages and dendritic cells and PD-L1 is expressed on tumor cells and other immune cells. Interaction of these ligands with PD-1 inhibits T-cell activation and cytokine production and is important in maintaining homeostasis of the immune response, thus preventing autoimmunity during infection or inflammation in normal tissue. The same interaction in TMEs provides an immune escape mechanism for tumor cells by turning off cytotoxic T cells. Blocking these interactions may enable the cytotoxic T cells in attacking the tumor cells. PD-L1 is a novel transcription target of HIF2α and HIF1α in tumor cell deficient in VHL [89, 90]. Blockade of HIF1α enhances myeloid derived suppressor cells (MDSC)-mediated T-cell activation. An unstable TME is associated with an increased expression of PD-L1 in tumor cells [93]. Inhibition of PD-L1 leads to an improved treatment outcome [87, 121]. PD-1 promotes ERK and mTOR pathways, and inhibition of the PD-1/PD-L1 axis leads to an enhanced antitumor activity of Docetaxel and Doxorubicin in an orthotopic metastatic mouse model [122]. PD-1/PD-L1 and HIFs are also associated with altered expression of specific types of oncogenic and tumor suppressor miRNAs. PD-L1 and FoxP3 regulatory T-cell infiltration of tumor cells are independent prognostic factors associated with poor prognosis in cancer patients [123, 124]. HIF1α mediates immune adaptation through the AKT/ERK/VEGF axis [125]. miRNA 155 and miRNA 210 target multiple pathways involved in the regulation of the immune response [126–130].

Collectively, current data suggests that PD-L1 expression is a result of an altered expression of miRNA and HIF, biomarkers that regulate multiple pathways including those that result in an unstable TME and cause tumor drug resistance. It is currently unclear whether the collective or individual modulation of these biomarkers by Se is necessary for tumor cell sensitization to targeted and cytotoxic drug therapies.

### Plasma Se concentrations derived from MSC or SLM determined therapeutic in xenograft models can be achieved clinically without dose-limiting toxicity

Enhancement of antitumor activity of multiple anticancer drugs by MSC/SLM in several xenograft models have been found to be dependent on Se dose and schedule. In several studies, oral administration of 10 mg/kg/day MSC or SLM were sufficient in inhibiting HIF by greater than 80% and in enhancing anti-tumor activity of anticancer drugs [15–18] To verify that the optimal plasma Se levels achieved in xenografts treated with therapeutic doses and schedule of SLM can be achieved clinically without host toxicities, cancer patients were treated with escalating doses of SLM.

**Figure 4:**
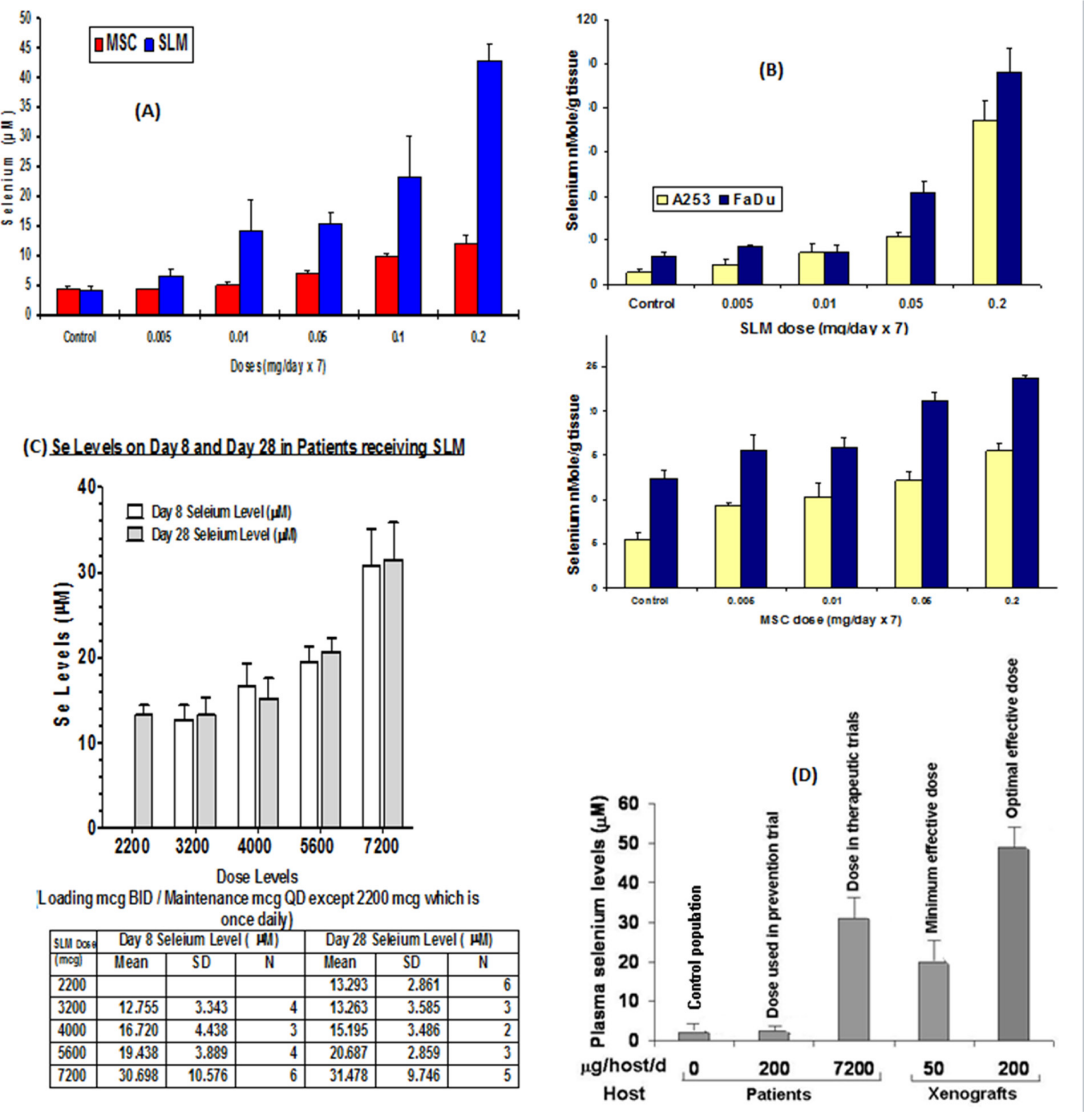
Plasma and tumor cells Se concentrations derived from the administration of therapeutic doses of MSC or SLM in mice and in plasma of patients treated with SLM **(A)** Total plasma Se level in mice after oral doses of MSC or SLM. **(B)** Intra tumor Se concentration 2h after 7 days of SLM or MSC [131]. **(C)** Plasma Se levels on day 8 and day 28 in patients receiving SLM [27, 28]. **(D)** Plasma Se levels in patients treated with SLM in the therapeutic trial reached levels higher than the minimal effective dose needed in mice for therapeutic augmentation with anticancer drugs and is significantly higher than the Se levels in untreated population or with SLM dose used in the prevention trial [35, 40].

The data in Figure 4A represents the total plasma Se levels achieved at 2h following the oral administration of different doses of MSC and SLM to xenografts. Minimal and optimal therapeutic doses for MSC and SLM were 0.05 mg, and 0.2 mg, respectively. Se plasma concentrations were dose dependent, with higher levels seen with SLM. Plasma Se concentrations after administration of 0.2 mg MSC and SLM orally were approximately 15 and 45 μM, respectively. Tumor tissue Se concentrations were also found to be Se dose-dependent (Figure 4B). As with plasma, A253 and FaDu tumors accumulated higher levels of Se derived from SLM than from MSC. Human studies were carried out in colorectal cancer patients treated with escalating doses of SLM in sequential combination with a fixed dose of irinotecan at a dose of 125 mg/m^2^/week for 4weeks, [27, 28]. The data in Figure 4C shows that the total plasma Se concentrations were dose-dependent. The dose of SLM in the prevention clinical trial, 200 μg/pt/d, yielded 3-4μM total Se plasma concentrations, slightly higher than the circulating levels in “normal” U.S. population. The dose of SLM at 7,200 μg/BIDx7 followed by 7200 μg/d concurrently with irinotecan for at least 28 days yielded plasma Se concentrations in the range of 30-40μM (Figure 4D), without toxicity. This concentration was similar to what was achieved with therapeutic doses of SLM in mice bearing human tumor xenografts.

The data in Figure 4D represents comparative levels of total plasma Se in a “control” population, patients in prevention trials, colorectal cancer patients treated with SLM, and in mice bearing tumor xenografts treated with the minimal and optimal therapeutic doses of SLM. Toxicity was limited to garlic breath in a few patients and minor dyspepsia [27, 29]. The clinical use of the high doses of SLM determined clinically safe is now under evaluation in sequential combination with axitinib, a VEGFR inhibitor, in heavily pretreated patients with advanced ccRCC.

Data presented in Table 3 is an attempt to correlate plasma and tumor tissue levels of Se with treatment outcome of xenografts with MSC or SLM in sequential combination with 100 mg/kg/wkX4 irinotecan (MTD) The data in Table 3 indicates that at a mouse plasma Se concentration of 10 μM derived post-administration of MSC, the tumor concentrations of SN38, the active metabolite of irinotecan, is higher in FaDu than in A253 xenografts. Cures, defined by the 90-day survival of xenografts without any evidence of cancer at the site of tumor transplant, seem to correlate with tumor levels of SN38. FaDu tumors with higher SN38 accumulation than A253, achieved a 100% cure rate compared with 60% cures seen in A253. Protection of xenografts from drug-induced toxicity by MSC allowed the administration of higher doses of irinotecan than the MTD that resulted in higher cures. While plasma and tumor tissue Se levels achieved with SLM were higher than those derived from MSC, a similar augmentation of the antitumor activity of irinotecan was seen in FaDu and A253 xenografts. The data in Table 3 also indicate that the higher cure rates of FaDu xenografts were associated with greater tumor cell concentrations of Se and SN38.

**Table 3:** Plasma and tumor Se levels achieved with oral aministration of 0.2gm/day MSC or SLM; correlation with “cure”, and tumor SN38 concentrations in A253, and FaDU xengrafts treated with irinotecan, 100 mg/kg/wkx4

Se (0.2 mg)	Plasma C_max_ (μM)	Tumor Se (nmol/gm)		“Cures” (%)		SN38 (nmol/gm)	
		A253	FaDU	A253	FaDU	A253	FaDU
**MSC**	15	16	22	60	100	13.1	29.4
**SLM**	48	80	100	60	100	ND	ND

In six patients with advanced colorectal cancer treated with 2200 μg SLM in combination with 125 mg/m^2^ irinotecan, no grade 3 diarrhea was observed and one out of six patients developed grade 4 neutropenia [27] with no grade 3 diarrhea. Normally, with the 125 mg/m^2^ per week irinotecan treatment, 20-30% of patients develop grade 3/4 diarrhea.

The optimal 0.2 mg/kg SLM dose delivered to xenografts was about 4000- and 57-fold higher than that achieved with 200 dose delivered to patients in the prevention clinical trial, and the 7,200 μg dose administered twice daily to cancer patients (assumed patient weight was averaged to approximately 85 kg), respectively. The SLM dose administered in our therapeutic clinical trial was about 70-fold higher than the dose used in the prevention trials. The high SLM dose utilized in our clinical trials, however, was 6-fold lower than the 40,800 μg dose of inorganic selenite that was determined to be toxic [25]. It is important to note that oral administration of 0.2 mg/kg MSC or SLM alone for several weeks resulted in minimal antitumor activity of less than 50% tumor growth inhibition. Doses lower than 0.05 mg/kg did not alter significantly the efficacy or the toxicity of irinotecan in the xenografts evaluated in the preclinical studies.

In summary, plasma Se concentrations in xenografts and in patients are Se dose-dependent. Similarly, enhanced antitumor activity of anticancer drugs in xenografts was MSC and SLM dose-dependent. Plasma Se concentrations associated with enhanced antitumor activity of anticancer drugs seen in mice bearing human tumor xenografts can be achieved clinically in human patients without toxicity. Enhanced tumor accumulation of Se and SN38 results in an enhanced antitumor activity of irinotecan

### Phase I clinical trial development

We have provided the rationale for use of a defined dose and schedule of SLM that can effectively enhance the therapeutic efficacy and selectivity of anticancer drugs [6–8, 15, 16], including axitinib, a tyrosine kinase inhibitor [19] in ccRCC xenografts. While MSC is under development, SLM is FDA-approved for clinical trials in the U.S. Patients with metastatic ccRCC who progressed on a prior line of treatment are being enrolled on 3 + 3 standard clinical trial design, combining escalating high doses of SLM at 2500, 3000, 4000, and 5000 μg in sequential combination with a standard of care axitinib dose. SLM is being given twice daily for 14 days, followed by once daily in combination with axitinib until disease progression or toxicity (NCT02535533). The plan is to develop a phase 2 clinical trial once the desired optimal plasma Se concentration has been achieved without toxicity as defined in the ongoing phase 1 trial

### Future directions

*In vivo* drug resistance is regulated by multiple molecular and immunological biomarkers and pathways expressed in tumor cells, the surrounding TME and cross-talk between them. Cells in the TME are functionally interactive, expressing different drug resistant biomarkers including specific types of oncogenic miRNAs, HIFs, VEGF/VEGFR, and PD-1/PD-L1, among others. While TME acts as a gatekeeper, tumor cells are the ultimate therapeutic targets. The molecular and immunological biomarkers implicated in angiogenesis and drug resistance can be modulated by therapeutic doses and schedules of MSC and SLM in several *in vitro* and *in vivo* models. The pleiotropic effects of SLM and MSC led to a significant enhancement of the antitumor activity of multiple anticancer drugs in several xenograft models. In addition to the demonstrated therapeutic benefits and protective effects against drug toxicities, MSC shares two unique properties: it downregulates expression levels of oncogenic miRNAs, include miRNA-155, −106b, and −210, and upregulates expression levels of tumor suppressor miRNAs, include miRNA-let 7b, −185, and −328; 2.

**Figure 5:**
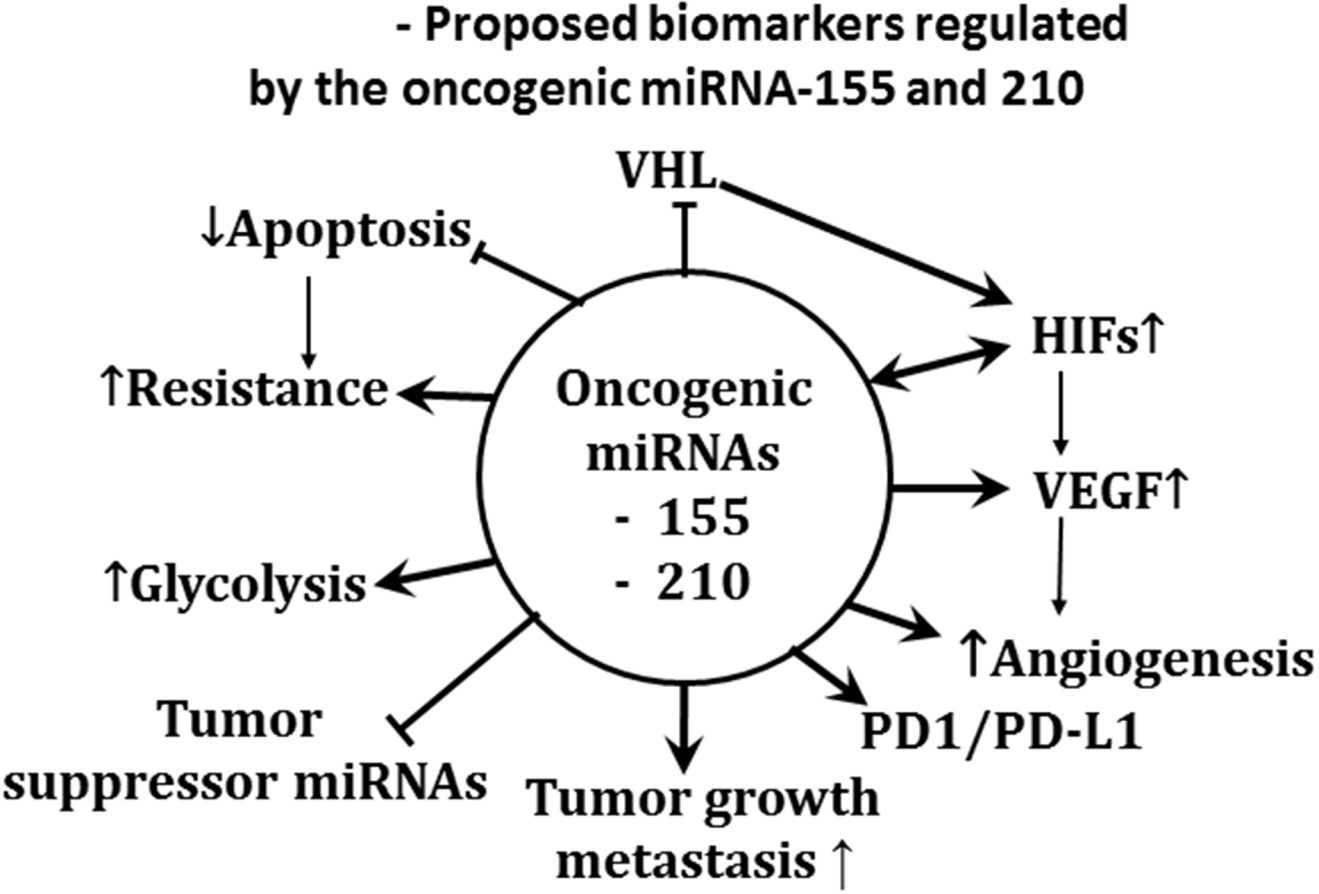
Pleiotropic effects of oncogenic miRNA-210, and miRNA-155 The proposed biomarkers modulated by the oncogenic miRNAs were identified from results generated primarily in RC2, 786.0, and primary ccRCC tumors, and, FaDu head and neck tumors

MSC also enhances degradation of hypoxia- induced and constitutively expressed HIF1α and HIF2α through PHD-dependent mechanism [8, 16]. Altered miRNAs and HIFs represent master regulators of angiogenesis, tumor growth and drug resistance. Based on the data generated in our laboratory, primarily in ccRCC tumors, together with published reports by others, the schema in Figure 5 represents the potential role of miRNA-155, and −210 in the modulation of multidrug resistance targets of Se. While the oncogenic miRNA regulates HIFs, these two miRNAs may interact and possibly regulate each other. From the therapeutic viewpoint, agents under development to target these markers have fallen short of clinical expectations and their limited clinical benefits have been attributed in part to their *in vivo* instability, toxicity, and inability to effectively modulate their intended targets [88]. Our laboratory was the first to demonstrate that the *in vivo* expression of HIFs and specific types of miRNAs altered in ccRCC tumor can be selectively modulated by a defined dose and schedule of Se. Recent data suggests that specific types of miRNAs in ccRCC tumors are regulated by HIF through VHL–dependent and independent pathways while PD-L1 is a target of the VHL-HIFs axis [121]. Thus, the reported stable expression of HIFs in ccRCC tumors may be a consequence of VHL inactivation by miRNA-155 and or miRNA-210.

To validate the concept that HIFs and specific types of miRNAs are critical therapeutic targets of Se whose modulation can impact treatment outcome, a pilot phase 1 clinical trial with SLM in sequential combination with axitinib is underway in pretreated patients with advanced ccRCC. Based on the results obtained in the pilot trial, a phase 2 clinical trial with SLM in sequential combination with other targeted agents will be developed.

### Overall summary

With the knowledge gained in our understanding of the pharmacology and the mechanism of action of cytotoxic drugs combined with the specific information and identification of biomarkers associated with angiogenesis and drug resistance, we have a unique opportunity to design new and novel mechanism-bases combinations. To this end, it is important to recognize that the dose, schedule and sequence of the drugs used in combination settings are critical in the design of future clinical trials. The unique profile of ccRCC tumors expressing mutant VHL stable expression of HIFs, and upregulated oncogenic miRNAs, and downregulated tumor suppressor miRNAs make it an excellent model for proof-of-concept that modulation of the expression levels and functions of these markers by therapeutically effective doses and schedules of Se may offer the potential to circumvent drug resistance, and may offer a new and novel clinical approach for the treatment of cancer
